# Functional disruption of stress modulatory circuits in a model of temporal lobe epilepsy

**DOI:** 10.1371/journal.pone.0197955

**Published:** 2018-05-24

**Authors:** Aynara C. Wulsin, Ana Franco-Villanueva, Christian Romancheck, Rachel L. Morano, Brittany L. Smith, Benjamin A. Packard, Steve C. Danzer, James P. Herman

**Affiliations:** 1 Department of Psychiatry and Behavioral Neuroscience, University of Cincinnati School of Medicine, Cincinnati, Ohio, United States of America; 2 Department of Anesthesia, Cincinnati Children Hospital Medical Center, Cincinnati, Ohio, United States of America; University of Modena and Reggio Emilia, ITALY

## Abstract

Clinical data suggest that the neuroendocrine stress response is chronically dysregulated in a subset of patients with temporal lobe epilepsy (TLE), potentially contributing to both disease progression and the development of psychiatric comorbidities such as anxiety and depression. Whether neuroendocrine dysregulation and psychiatric comorbidities reflect direct effects of epilepsy-related pathologies, or secondary effects of disease burden particular to humans with epilepsy (i.e. social estrangement, employment changes) is not clear. Animal models provide an opportunity to dissociate these factors. Therefore, we queried whether epileptic mice would reproduce neuroendocrine and behavioral changes associated with human epilepsy. Male FVB mice were exposed to pilocarpine to induce status epilepticus (SE) and the subsequent development of spontaneous recurrent seizures. Morning baseline corticosterone levels were elevated in pilocarpine treated mice at 1, 7 and 10 weeks post-SE relative to controls. Similarly, epileptic mice had increased adrenal weight when compared to control mice. Exposure to acute restraint stress resulted in hypersecretion of corticosterone 30 min after the onset of the challenge. Anatomical analyses revealed reduced Fos expression in infralimbic and prelimbic prefrontal cortex, ventral subiculum and basal amygdala following restraint. No differences in Fos immunoreactivity were found in the paraventricular nucleus of the hypothalamus, hippocampal subfields or central amygdala. In order to assess emotional behavior, a second cohort of mice underwent a battery of behavioral tests, including sucrose preference, open field, elevated plus maze, 24h home-cage monitoring and forced swim. Epileptic mice showed increased anhedonic behavior, hyperactivity and anxiety-like behaviors. Together these data demonstrate that epileptic mice develop HPA axis hyperactivity and exhibit behavioral dysfunction. Endocrine and behavioral changes are associated with impaired recruitment of forebrain circuits regulating stress inhibition and emotional reactivity. Loss of forebrain control may underlie pronounced endocrine dysfunction and comorbid psychopathologies seen in temporal lobe epilepsy.

## Introduction

Temporal lobe epilepsy (TLE) is the most prevalent form of refractory epilepsy in adults, accounting for the majority of epilepsy patients requiring neurosurgery [1–3]. In some instances, TLE is preceded by an initial brain insult such as status epilepticus (SE), febrile seizures or trauma [4]. Such injuries can precipitate the development of aberrant networks in the hippocampus and other limbic structures [5,6]. Limbic network reorganization is directly implicated in the generation of seizure activity, as is evident from the efficacy of temporal lobectomy in controlling seizures [1]. In addition to its role in seizures, however, limbic reorganization may also disrupt neuroendocrine processes and emotional behaviors, contributing to the development of highly comorbid stress-related psychopathologies [7–9].

The hypothalamo-pituitary-adrenocortical (HPA) axis regulates somatic responses to both physical and emotional stress. Efficient activation and effective termination of the response is tightly regulated by negative feedback effects of glucocorticoids acting at numerous CNS loci, including the hypothalamus, prefrontal cortex, hippocampus and amygdala [10–12]. Thus, intact limbic networks are required for optimal HPA axis control. Chronic elevations in glucocorticoids are observed in a subset of patients with TLE [13], consistent with enhanced HPA axis activation and/or reduced feedback signaling. In addition, cortisol stress responses can be exaggerated in some TLE patients, and such enhanced reactivity is associated with reduced functional connectivity between the hippocampus and the frontal cortex [14]. Importantly, HPA axis hyperactivity and consequent elevations in glucocorticoids are known to remodel [15] and potentially injure [16] limbic networks while also predisposing individuals to stress-related psychopathologies [17–19]. Thus, limbic reorganization may occur at multiple levels in TLE, as a result of the initial precipitating injury, as a consequence of repeated seizure activity and following excess glucocorticoid exposure. Reorganization may result in abnormal responses to stress and increased susceptibility to aberrant emotional behaviors.

The pilocarpine-induced SE model of temporal lobe epilepsy has been extensively utilized to investigate mechanisms of epileptogenesis [3,20–22]. Additionally, we and others have shown that the initial SE leads to increased baseline secretion of glucocorticoids [23,24] that is likely to persist through the chronic epileptic period [25]. The purposes of this study are: 1) to assess the endocrine response to acute stress in the pilocarpine TLE model and 2) to determine whether epilepsy compromises the recruitment of key stress regulatory regions. Additionally, we aim to assess the validity of pilocarpine-induced SE as a model to study the complex relationship between HPA axis dysfunction and susceptibility to psychopathologies observed in human TLE.

## Materials and methods

### Animals

Forty 8-week-old male FVB mice (Charles River International Strain Code 207) were housed individually in standard mouse shoebox cages and acclimated to laboratory conditions for 2 weeks prior to all experimental procedures. Mice were maintained in a temperature and humidity controlled room (lights on 0500–1700) with food and water available *ad libitum*. All experimental procedures and protocols were conducted as approved by the University of Cincinnati Institutional Animal Care and Use Committee and in accordance with NIH guidelines for the Care and Use of Animals. Protocols were designed to limit animal stress and discomfort.

### Pilocarpine protocol and experimental design

**Fig 1:**
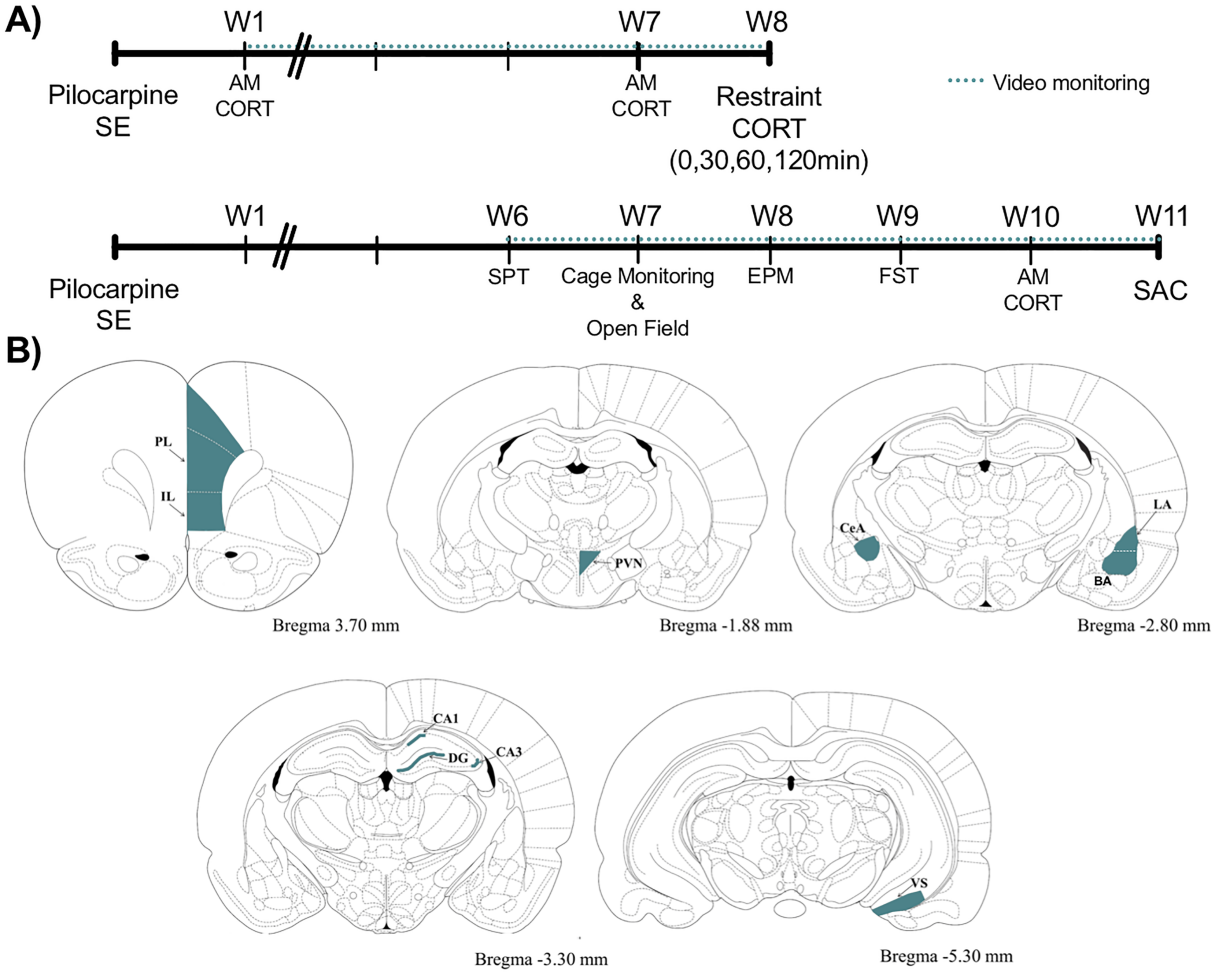
Experimental design. A) Experimental time line for cohorts 1 (top) and 2 (bottom). Baseline morning corticosterone (CORT) samples were taken at 1 and 7 weeks post-SE for Cohort 1 and at week 10 for Cohort 2. At week 8, mice from Cohort 1 were exposed to restraint and blood samples were taken at 0, 30, 60, and 120 minutes from the onset of the stressor. Cohort 2 mice began behavioral testing at week 6 (SPT, sucrose preference test; EPM, elevated plus maze; FST, forced-swim test). B) Brain regions analyzed for Fos expression are based on the Paxinos and Watson brain atlas. Abbreviations. IL, infralimbic prefrontal cortex; PL, prelimbic prefrontal cortex; hippocampal subdivisions include: CA1, CA3, DG, dentate gyrus; VS, ventral subiculum; amygdaloid complex: CeA, central amygdala; LA, lateral amygdala; BA, basal amygdala and PVN, paraventricular nucleus of the hypothalamus. Figure is modified from [26].

Pilocarpine is a muscarinic 1 (M_1_) receptor agonist that has been used extensively to induce seizures in rodents ([3] for review). High doses of pilocarpine induce a state of persistent seizures (SE) lasting many hours, followed by the development of spontaneous seizures weeks later. For the present study, all mice were given a subcutaneous injection (s.c.) of methyl scopolamine nitrate (1 mg/kg) followed by pilocarpine (350 mg/kg, s.c.) or saline (s.c.) 15 min later. Experiments were run in two randomly-generated cohorts, each with 9–10 saline and 10–11 pilocarpine-treated mice. Mice were monitored behaviorally for seizure activity and the onset of status epilepticus (SE) was noted as previously described [23]. After 3 hours of SE, all mice—including saline controls—received two injections of diazepam 10 min apart (10 mg/kg, s.c.) to mitigate seizure activity. For the next 48h mice were given sterile Ringer’s (saline) solution 2–3 times daily for hydration support and access to soft diet. Mice were video-monitored throughout the entire experiment. Body weight was assessed once weekly in all mice until the end of the experiment. To confirm the presence of spontaneous seizures, 3 days of video data collected in the 6th week after SE were reviewed. Seizures were classified as either Class II/III or IV/V using a modified Racine scale [26]. In the present experiment 10% of pilocarpine-treated mice died during SE and another 14% died (or were euthanized due to declining health) over the following days. Overall mortality among pilocarpine-treated mice was 24% (5 of 21). No control mice died. Final cohorts were as follows: *Cohort 1* (n = 7 SE mice, n = 9 Controls) *Cohort 2* (n = 9 SE mice, n = 10 Controls). All 16 pilocarpine-treated mice had at least one Class V spontaneous seizure [Cohort 1: 9.7±4.4 seizures/day, Cohort 2: 6.6±2.1]. *Cohort 1* was used for the assessment of HPA axis function and *Cohort 2* was used for behavioral testing (Fig 1A).

### HPA axis assessment

Morning baseline corticosterone levels were obtained by collecting tail-nick blood 1 and 7 weeks after SE in cohort 1 mice and week 10 in mice of cohort 2. Eight weeks after SE, mice were exposed to restraint stress for 30min. For restraint stress, mice were placed in well ventilated conical tubes for 30min. Plasma samples were collected by tail nick at 0 (pre-stress), 15, 30, 60 and 120min after mouse placement in the restrainer. The 30min sample was collected while animals were inside the restrainer. All other plasma samples, including baseline measurements, were collected from freely-moving mice using a procedure that can be completed in under 3 min (the minimal response time for HPA axis activation in rodents [27]), to minimize stress [28]. Immediately after the 120min plasma collection, mice were given pentobarbital (100 mg/kg i.p) and transcardially perfused with saline followed by 2.5% paraformaldehyde + 4% sucrose in 0.1M PBS. Brains were collected and sectioned on a microtome at 35 μm for immunohistochemistry.

Mice that exhibited a seizure up to 6 hours prior to or during restraint stress were excluded, in accord with past work suggesting that 6 hours is sufficient time to eliminate the confounding effect of acute seizures on corticosterone secretion [25]. Assessment of acute seizures was conducted using a combination of real-time observations by the experimenter and retrospective video analyses. Only one mouse exhibited a class V seizure during restraint stress. In addition, two 7-week samples were removed from cohort 1 and one baseline sample was removed from cohort 2 due to presence of seizure activity.

#### Corticosterone radioimmunoassay

Tail blood samples (~50μl) were collected into EDTA-treated microvette collection tubes and immediately placed on ice. All blood sample collections occurred within 4 h of lights on. Blood samples were centrifuged (6000 g, 15min, 4°C) to isolate the plasma, which was subsequently stored at -20°C. Plasma corticosterone levels were measured using 125I RIA kit (MP Biomedicals Inc., Orangeburg, NY) as previously described [23].

### Organ collection

In order to assess the impact of epilepsy on stress-sensitive organs, adrenal and thymus glands from mice in cohort 2 were dissected, blotted on filter paper, allowed to dry and immediately weighed.

### Behavioral assessment

6 weeks after SE, mice in cohort 2 underwent the sucrose preference test. The following week, 24h home cage monitoring was conducted, followed 72h later by open field testing. On week 8 mice were tested in the elevated plus maze, followed by testing in the forced swim test 9 weeks after SE. The open field, elevated plus maze and forced swim tests were performed between 09:00–12:00 under regular room lights.

#### Sucrose preference test

Sucrose preference, a measure of anhedonia, was assessed using singly-housed mice as previously described [29]. Mice were allowed to acclimate for 3 days to two water bottles. On the fourth day, the water in one of the bottles was replaced with sucrose solution (0.5%) for 24h, followed by an additional 24h period with water-only in each bottle. Beginning on the sixth day, a 48h sucrose preference test was performed by allowing mice to choose between bottles with either 1% sucrose or water. The bottles were reversed on the second day to control for side preference. Intake from each bottle and body weight were measured daily. Sucrose preference was calculated as the ratio of sucrose solution consumed divided by total fluid consumption over the 48hr period.

#### Home-cage activity monitoring

Twenty-four hour home cage activity was measured using the SmartFrame stainless steel cage rack frame (Lafayette Instrument Company, Lafayette, IN, USA) with infrared photo beam interruption sensors (7X and 15Y). Data were analyzed using HMM100 motor monitor software (Campden Instruments LTD, England) [30].

#### Open field

Mice were exposed to an open field apparatus consisting of a white 50 X 50 X 22 cm Plexiglas box. Each mouse was placed in the same initial corner facing the walls and allowed to freely explore for 5 min. The single testing session was video recorded. Videos were scored for total time spent in the periphery and center areas as well as distance travelled using Topscan software (Clever Sys Inc, Reston VA, RRID:SCR_014494). Behavioral freezing was analyzed using EthoVision XT software (Noldus, VA, RRID:SCR_000441).

#### Elevated plus maze

Exposure to the elevated plus maze was used as a measure of innate anxiety. The PVC apparatus consists of a 5 cm^2^ center area from which two open (66 × 5 cm) and two enclosed (66 x 5 × 15 cm) arms radiate. Arms were 38 cm above the floor. For testing, each mouse was placed on the center square of the maze facing the same open arm. Video files were scored using Topscan software (Clever Sys Inc, Reston VA, RRID:SCR_014494) for time spent in the open vs. closed arms and total distance travelled. Freezing behavior was determined using EthoVision XT software (Noldus, VA, RRID:SCR_000441).

#### Forced swim test

Mice were exposed to a single 6 minute session as previously described [26]. The behavioral apparatus was a 2 L glass beaker filled with 1500 ml of water (30–33°C). Mice were placed in the cylinder for 6 min and the session was videotaped. Scoring was done by an observer blind to the experimental conditions. Behavior was scored by quantifying the time spent in (i) active struggling behaviors, defined as any movement of the limbs in and out of the water with the body parallel to the surface or as circular movement around the apparatus, and (ii) immobility, defined as periods when the mouse did not make any active movements or floated in the water without struggling.

### Immunohistochemistry

Free-floating brain sections (35 μm) were placed in blocking solution (4% normal goat serum, 0.1% bovine serum albumin and 0.2% Triton-X in 0.1 M PBS) for 1h and subsequently incubated in rabbit anti-Fos (1:500 Santa Cruz Biotechnology Cat# sc-52, RRID: AB_2106783) and mouse anti-NeuN (1:500 Millipore Cat# MAB377, RRID:AB_2298772) antibodies for 24h at 4°C. Both primary antibodies are well-characterized [31,32] and have been used previously by our group [26,33,34]. Donkey anti-rabbit AlexaFluor 594 (1:500 Jackson ImmunoResearch Labs Cat# 711-585-152 RRID:AB_2340621) and goat anti-mouse AlexaFluor 488 (1:500 Jackson ImmunoResearch Labs Cat# 115-545-146 RRID:AB_2307324) antibodies were used to visualize the respective primary antibodies. Stained tissue sections were mounted onto gelatin-coated slides and coverslipped with Fluoromount-G mounting media. Three to four sections per animal were examined for each of the regions summarized in Fig 1B. Because acute seizures have been shown to increase Fos expression in the hippocampus [35], the one mouse that was excluded from the restraint testing due to a seizure was also excluded from immunohistochemical assessment.

### Confocal imaging and cell quantification

Images were obtained using a 3024 Nikon A1Rsi microscope (software RRID: SCR_014329) with a 40× water objective (NA 1.15, image size 317 × 317 μm in the XY plane). All images were collected with identical parameters and analyzed by an investigator blind to the experimental conditions to avoid bias. Three to four sections per brain region were analyzed. The regions analyzed (Fig 1B) were selected based on their known role in regulating the HPA axis response to stress [12,36,37]. We analyzed Fos activation in two areas of the medial prefrontal cortex (infralimbic and prelimbic), three subdivisions of the hippocampus (CA1, CA3 and dentate gyrus), three amygdala subdivisions (central, basal and lateral amygdala), the ventral subiculum and the paraventricular nucleus of the hypothalamus (Fig 1B). The proportion of Fos to NeuN positive cells in each region was determined from confocal z-series image stacks (0.33 μm step size through ~10 μm of tissue). All cell counts were conducted using Imaris Software (RRID: SCR_007370) and a variation of the optical dissector method [38,39]. Fluorescent “spots” automated detection method [40] was used to determine the number of immunopositive cells. A volume surface was created by tracing the region of interest (ROI) through the stack. Minimum fluorescent intensity and size criteria (>4.14μm) were used for automated screening. The number of Fos and NeuN immunopositive cells were further reviewed by an investigator blinded to treatment group to remove false-positives and to identify false-negatives. Results are reported as percentages of total Fos positive cells over total NeuN positive cells to correct for any cell death.

### Statistics

Statistical analyses were performed using GraphPad Prism software (version 7.0) or SigmaPlot Software (version 13.0). Unpaired t-test’s with Welch’s correction were performed to compare single measurements between epileptic and control mice. Statistical significance for all immunohistochemical analyses was determined using Sidak-Bonferroni corrections for multiple t-tests. 2 –Way Repeated Measures ANOVA with Holm-Sidak post-hoc analysis was employed to analyze corticosterone measurements following restraint stress, baseline secretion from cohort 1 and home cage monitoring of cohort 2 data. Statistical significance was set at p ≤ 0.05. Data that failed tests of normality or equal variance were normalized using either square root or rank transformations. Values are presented as means of raw (non-transformed) data ± standard error of the mean (SEM).

## Results

### Epileptic mice show elevated stress-induced and baseline corticosterone secretion

**Fig 2:**
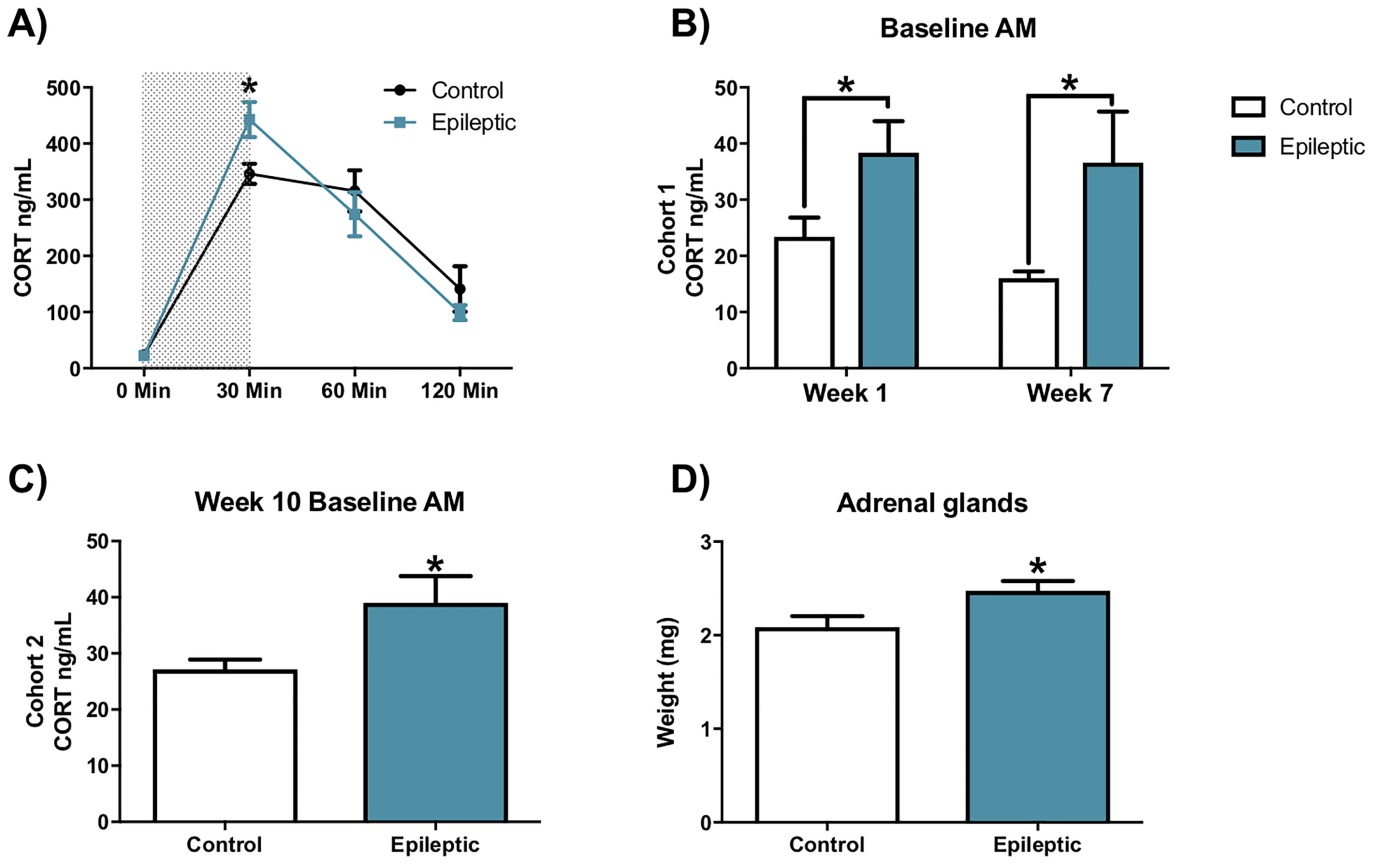
Corticosterone hyper-secretion is present at baseline and stress-induced states in epileptic mice. A) Epileptic mice show increase levels of corticosterone (CORT) secretion following 30 minutes of restraint stress (gray region shows time in restrainer). B) Epileptic mice in cohort 1 show increased corticosterone baseline secretion at 1 and 7 weeks compared to control mice. C) Epileptic mice in cohort 2 show increased corticosterone baseline secretion at week 10 relative to controls. D) Adrenal weight of epileptic mice in cohort 2 is greater than control mice. (*p < 0.05, epileptic vs. control; ***p < 0.001). Data presented as mean ± SEM, Cohort 1 n = 6–8 mice per group, Cohort 2 n = 7–9 mice per group).

Eight weeks after SE, mice from cohort 1 were exposed to restraint stress for 30 minutes. Blood samples to analyze corticosterone secretion were taken at 0, 30, 60 and 120 min from the onset of the stressor. Restraint produced a significant increase in corticosterone levels in both control and epileptic mice (Fig 2A; F[3,34] = 86.73, p<0.001, Two-way RM ANOVA with timepoint and treatment as factors). Relative to pre-restraint levels (0 minutes) corticosterone was significantly increased at 30 minutes (p<0.001, Holm-Sidak method) and 60 minutes (p<0.001, Holm-Sidak) for both treatment groups. In addition, a significant interaction between timepoint and treatment was found (F[3,34] = 3.59, p<0.023). Post-tests revealed a significantly greater corticosterone increase at the 30 minute timepoint in epileptic mice relative to controls (p<0.016, Holm-Sidak). Control and epileptic mice were statistically equivalent at 0 (p = 0.930), 60 (p = 0.205) and 120 (p = 0.235) minutes.

In addition to acute stress challenge, we also assessed baseline corticosterone levels. Morning baseline corticosterone secretion was elevated in epileptic mice compared to controls (Fig 2B; F[1,11] = 16.24, p = 0.002, Two-way ANOVA on ranked data with timepoint and treatment as factors]. Post-tests revealed significantly greater corticosterone levels in epileptic mice vs. controls at both 1 week (p = 0.02, Holm-Sidak) and 7 weeks (p<0.001, Holm-Sidak). Epileptic mice from cohort 2 also showed increased morning baseline corticosterone secretion when measured at week 10 (Fig 2C; t = 2.352 df = 8.79, p = 0.022, t-test). Consistent with these findings, adrenal gland weight from epileptic cohort 2 mice was significantly increased relative to controls (Fig 2D; t = 2.469 df = 15.73, p = 0.0127, t-test). Thymus weight did not differ between groups (data not shown).

### Reduced Fos expression in stress regulatory forebrain regions following restraint stress in epileptic mice

**Fig 3:**
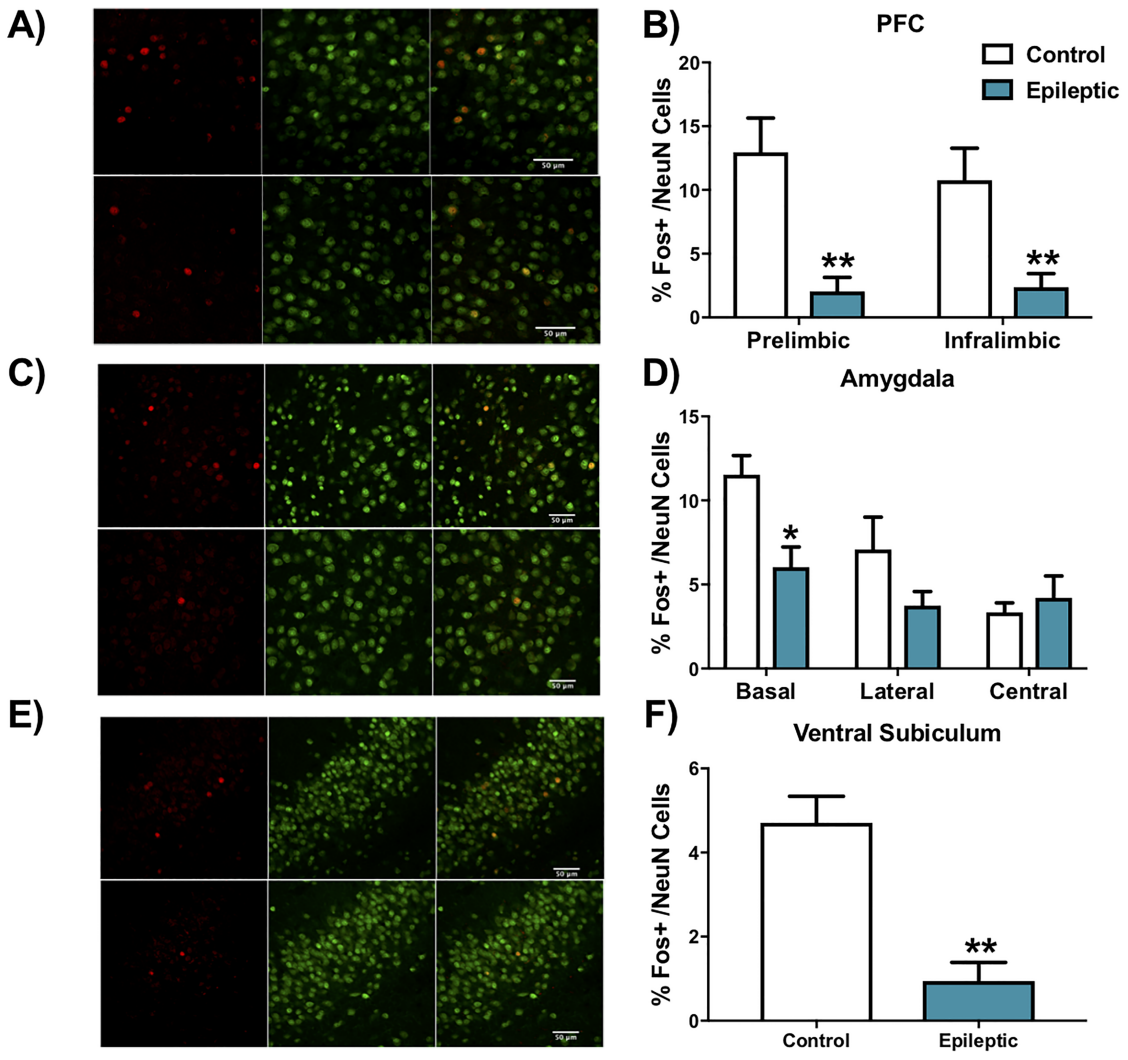
Reduced expression of Fos in stress regulatory regions in epileptic mice. Representative micrographs showing Fos (red) and NeuN (green) immunoreactivity in A) the prelimbic region of the medial prefrontal cortex (PFC), C) the basal amygdala and E) ventral subiculum. Top panels in each set show control mice while bottom panels are from epileptic mice. Epileptic mice show decreased Fos expression in the B) prelimbic and infralimbic region of the PFC, D) the basal subdivision of the amygdaloid nucleus and F) the ventral subiculum relative to control mice. *p < 0.05, **p<0.01. Data presented as mean ± SEM, n = 6–8 mice per group.

Restraint stress has consistently been found to increase Fos immunoreactivity in specific brain regions in control animals [26,33,41–47]. Consistent with these prior findings, control (non-SE) mice exposed to 30 minutes of restraint stress exhibited large numbers of Fos immunoreactive neurons in both subdivisions of the medial prefrontal cortex (PFC), measured 120 min following stress induction. By contrast, Fos immunoreactive neurons were rare in epileptic mice following stress (Fig 3A & 3B; prelimbic t = 3.356 df = 12, p = 0.01; infralimbic t = 2.731 df = 12, p = 0.03; t-test with Sidak-Bonferroni corrections). Similarly, there were significantly fewer Fos immunoreactive neurons in the basal amygdala (Fig 3C & 3D; t = 3.298 df = 12, p = 0.02; t-test with Sidak-Bonferroni corrections) and the ventral subiculum (Fig 3E & 3F; t = 4.56 df = 12, p = 0.003; t-test) in epileptic mice. No significant differences were found in the hippocampus, or other amygdaloid subnuclei (Table 1). Epileptic mice exhibited a significant reduction in NeuN+ cells in the CA3 region of the hippocampus (Table 1; t = 3.075 df = 12, p = 0.03), fitting the known pattern of SE-induced cell loss in this model. NeuN+ cell densities were statistically similar between epileptic and control mice for all other regions analyzed (Table 1).

**Table 1:** Fos expression quantification.

		NeuN+ Cells		Fos+ Cells		%Fos+/NeuN+ Cells	
		Control	Post-SE	Control	Post-SE	Control	Post-SE
PFC	PL	786±45	766±58	104±24	17±10*	12.96±2.86	2.05±1.19*
	IL	512±25	479±53	57±16	13±7*	10.76±2.68	2.38±1.16*
AMY	Basal	356±33	466±16	40±5	28±6*	11.53±1.20	6.03±1.31*
	Lateral	255±18	285±10	19±7	11±3	7.09±2.05	3.75±0.91
	Central	304±20	367±50	10±2	14±4	3.34±0.60	4.21±1.42
HIPP	CA1	188±5	180±14	2±1	2±2	1.12±0.31	1.14±0.86
	CA3	249±11	148±39*	10±1	5±2	4.14±0.51	3.78±1.16
	DG	1502±56	1426±75	23±6	8±2	1.47±0.35	0.56±0.15
Ventral Subiculum		504±10	513±27	24±3	5±3*	4.71±0.67	0.94±0.48*
PVN		300±14	283±26	134±14	108±4	44.39±3.53	39.19±3.16

Group averages showing the number of NeuN+ and Fos+ cells per region are shown in columns 2 and 3: The rows represent infralimbic (IL) and prelimbic (PL) medial prefrontal cortex (PFC); basal, lateral and central amygdala (AMY); CA1, CA3 and dentate gyrus (DG) of hippocampus (HIPP); ventral subiculum and paraventricular nucleus of the hypothalamus (PVN). To control for cell loss, the number of Fos+ cells was normalized to the number of NeuN+ cells and converted to a percentile (Fos+/NeuN+ X 100). Data are reported as means ± SEM. n = 6–8 mice per group.

*p < 0.05 epileptic different control.

### Epileptic mice display anxiety-like behaviors

**Fig 4:**
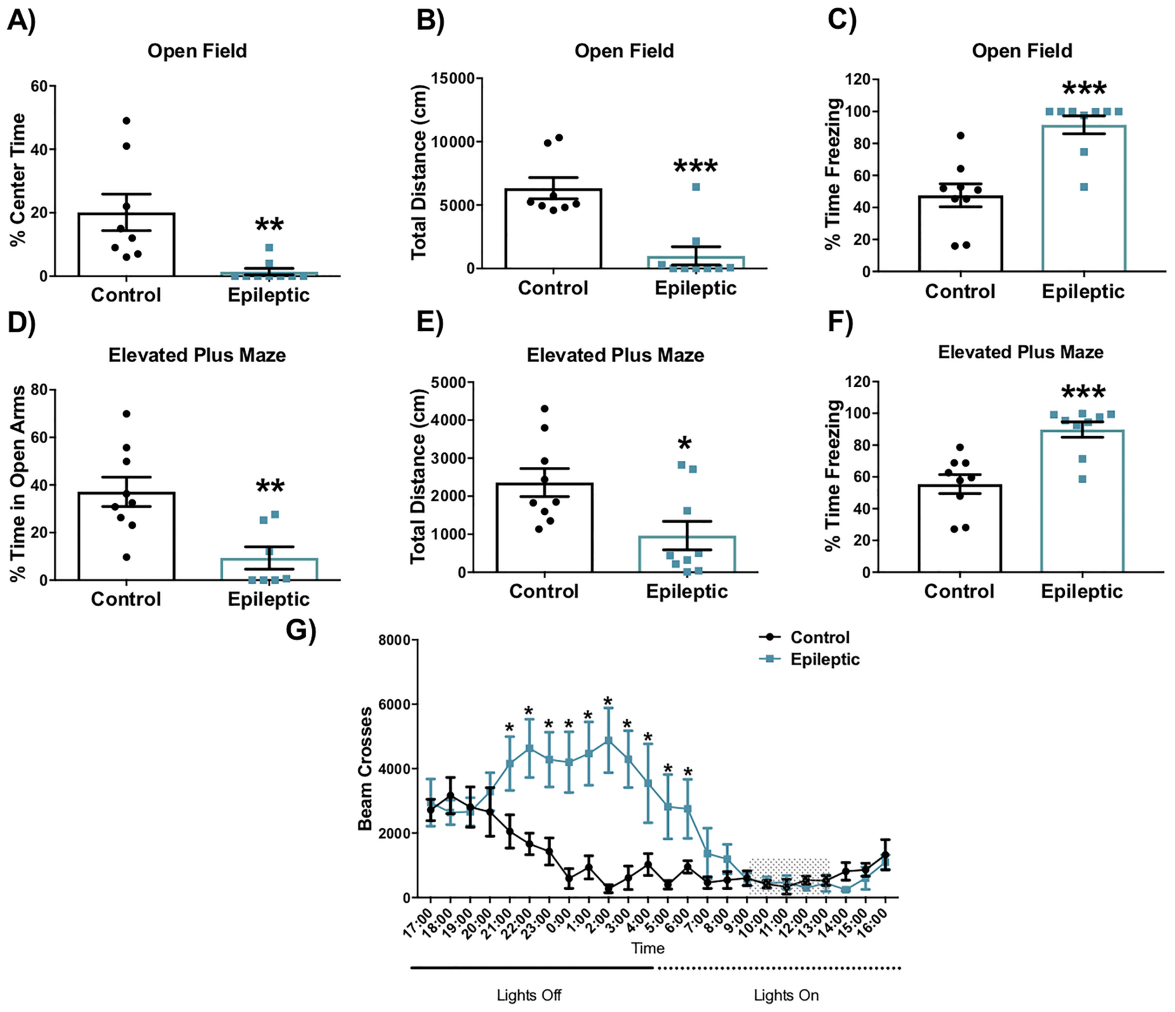
Epileptic mice show anxiety-like and hyperactive behaviors. Epileptic mice show reduced percent time spent in the center of the A) open field and D) open arms of the elevated plus maze. The total distance travelled by epileptic mice in the B) open field and E) elevated plus maze is decreased relative to controls. Epileptic mice spent more time freezing (immobility >2 seconds) in the C) open field and in the F) elevated plus maze compared to their control counterparts. G) 24h activity monitoring demonstrates overall hyperactivity of epileptic mice relative to control mice. Shaded region indicates the time when all behavioral tests were performed. (*p < 0.05, **p < 0.01, ***p < 0.001). Data presented as mean ± SEM, n = 9 mice per group.

Epileptic mice exhibited a significant reduction in the percentage of time spent in the center of the open field relative to controls (Fig 4A; t = 3.189 df = 7.458, p = 0.0141, t-test). Epileptic mice also displayed a reduction in the total distance travelled in the open field (Fig 4B; t = 4.842 df = 14.34, p = 0.0002, t-test) and exhibited significantly more freezing behavior, defined as the complete absence of movement—except for breathing—lasting >2s (Fig 4C; t = 4.846 df = 15.09, p = 0.0002, t-test). Similarly, in the elevated plus maze, epileptic mice avoided the open arms (Fig 4D; t = 3.592 df = 13.81, p = 0.003, t-test) and showed a reduction on the total distance travelled (Fig 4E; t = 2.648 df = 16, p = 0.0176, t-test). Epileptic mice also exhibited increased freezing behavior in the plus maze (Fig 4F; t = 4.449 df = 15.38, p = 0.0004, t-test), similar to that observed in the open field. Reduced movement in the maze trials, however, does not appear to reflect an overall decrease in mobility or ill health, as 24 hour home-cage activity monitoring revealed that epileptic mice were more active than controls. (Fig 4G; F[23,368] = 5.329, p<0.001; two-way ANOVA on ranked data showing significant interactions between timepoint and treatment). Post-tests revealed a significant increase in activity between 21:00–06:00 (p<0.001, Holm-Sidak) of epileptic mice relative to controls. Importantly, behavioral testing was done during the light phase, when epileptic and control mice displayed similar activity levels (09:00–13:00).

### Epileptic mice exhibit increased anhedonic behaviors, but not increased passive coping behaviors

**Fig 5:**
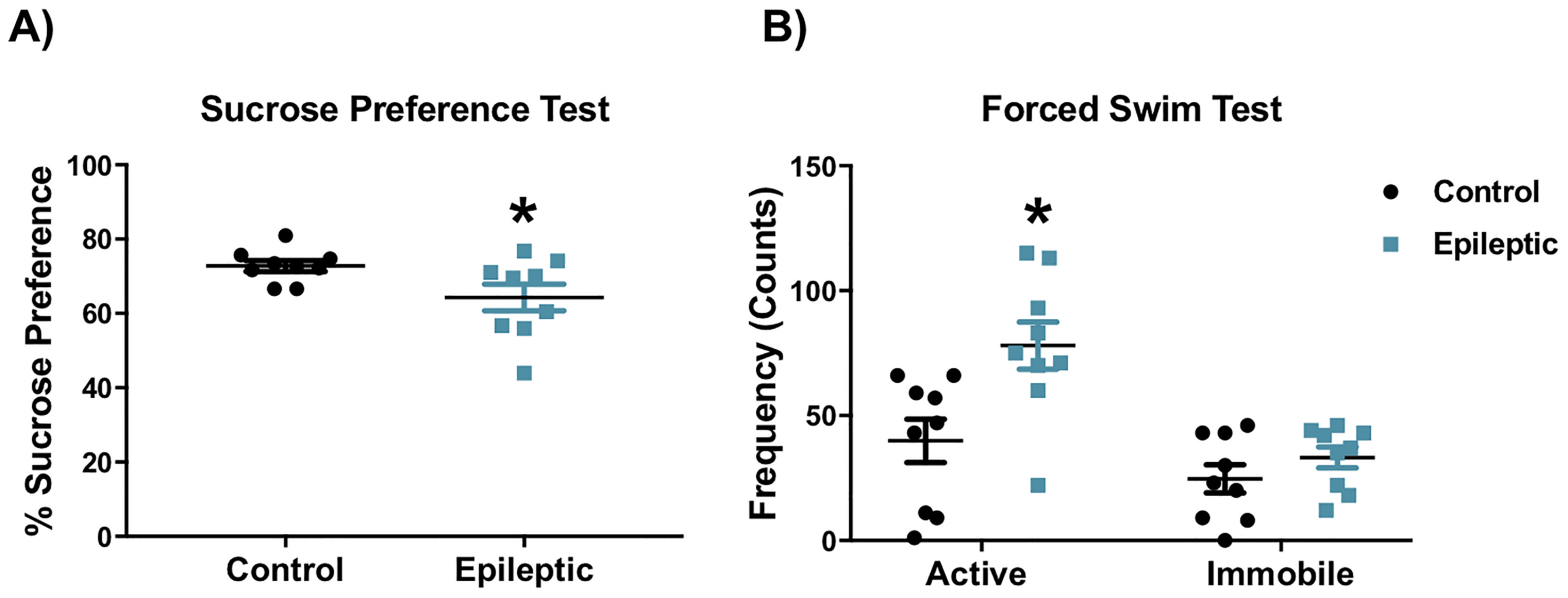
Epileptic mice show anhedonia and hyperactive behaviors. A) Sucrose preference was decreased in epileptic mice relative to controls. B) Epileptic mice showed increased active behaviors in the forced swim test. (*p < 0.05). Data presented as mean ± SEM, n = 9 mice per group.

The sucrose preference test was used as a measure of anhedonia, a behavior that is commonly observed in humans with major depressive disorder. Epileptic mice showed reduced preference for the 1% sucrose solution when compared to control mice (Fig 5A, t = 2.201 df = 10.65, p = 0.03, t-test). The forced swim test was used to assess coping behavior. Epileptic mice displayed more frequent active behaviors than control mice (Fig 5B, t = 2.976 df = 16, p = 0.017, t-test). No significant differences were observed in the percent of time spent immobile in the forced swim test.

## Discussion

Our results demonstrate that the induction of epilepsy in mice causes profound decreases in activation of key brain circuits that are responsible for the control of stress reactivity and emotional behavior. Loss of forebrain stress reactivity is associated with enhanced glucocorticoid secretion in response to acute stress. Importantly, pilocarpine-induced epilepsy also increases anxiety- and depression-like behaviors thought to be regulated by prefrontal and hippocampal circuits. Such findings are consistent with the increased incidence of anxiety and depressive disorders observed in patients with epilepsy, and suggest that epilepsy produces an organic brain dysfunction in key stress-regulatory circuits that may increase vulnerability for the development of psychological disorders.

### Endocrine dysregulation occurs during epileptogenesis and persists through the chronic epileptic period

Pilocarpine SE induces a rapid increase in glucocorticoid secretion, evident as early as 2 hours after SE in mice [24]. Here, we show that glucocorticoid hypersecretion is present 1 week after SE, before spontaneous seizures were observed, and 7 and 10 weeks after SE, when spontaneous seizures were frequent. The findings are consistent with previous reports [23,25]. Although the data suggest that glucocorticoid hypersecretion at 1 week may be a consequence of SE, we cannot exclude the possibility that increased secretion was driven by undetected spontaneous seizures. Additionally, we report an increase in adrenal gland weight at 10 weeks post-SE, indicative of increased adrenal drive.

Epileptic mice were hyper-responsive to acute restraint stress, showing elevated peak corticosterone secretion. This hyper-responsiveness is reminiscent of that observed in a subset of patients with TLE [13,14]. Damage to the hippocampus, either due to the initial period of status or recurrent seizures, may be responsible for the glucocorticoid hypersecretion seen in epileptic mice. A number of lesion studies implicate the hippocampus in inhibition of basal (circadian nadir) corticosterone secretion [37]. Hippocampal damage is characteristic in humans with temporal lobe epilepsy, and in animal models of the disease [3,48–50]. Loss of CA3 pyramidal cells was observed in the present study, predictive of disrupted hippocampal output. Indeed, glucocorticoids play an important role in regulating hippocampal neuronal viability following excitotoxic insults such as exposure to kainic acid [51]. Consequently, it is plausible that the observed increases in baseline morning corticosterone may be linked to pathological changes in the hippocampus of post-SE mice. However, SE has the capacity to affect the entire brain, and we cannot exclude the possibility that damage to other regions contributes to endocrine dysfunction.

### Stress modulatory forebrain circuits are disrupted in chronic epilepsy

Fos is the protein product of immediate early gene c-Fos which is expressed and detectable within minutes to hours following an acute cellular stimulus [52–54]. Under unstressed conditions, Fos expression is minimal [42,44,55]. However, exposure to acute stress produces robust increases in Fos protein in numerous brain regions, including the prefrontal cortex and the paraventricular nucleus of the hypothalamus in mouse and rat [26,33,41–47].

Strikingly, 30 minutes after restraint challenge, Fos protein levels in epileptic mice were significantly lower than controls in the medial prefrontal cortex, basal amygdala and ventral subiculum. We cannot directly compare induction seen in epileptic mice to those of unstressed controls, so it is not possible to conclude that these regions are inactive. However, it is clear that Fos reactivity is substantially less than that observed in stressed non-SE mice, consistent with disengagement of corticolimbic stress circuitry. Importantly, we are not aware of other SE-related mitigating factors that cause pronounced reductions in stress-induced Fos. Elevated Fos levels are evident 15 minutes after spontaneous seizures in the rodent pilocarpine model [35], but Barros and colleagues [56], report that Fos levels return to baseline by 6 hours after a pentylenetetrazole-evoked seizure. Exclusion of animals exhibiting behavioral seizures up to 6 hours before testing in the present study should minimize the effect of post-ictal changes in Fos levels, although it remains possible that the post-ictal influence on Fos extends beyond this time frame. Nevertheless, the fact that Fos is minimally induced in the prefrontal cortex, amygdala and ventral subiculum of epileptic mice is remarkable and unique, and suggests compromised integrity of stress-regulatory circuitry.

The recruitment of forebrain structures is thought to modulate activation of the HPA axis, providing top-down regulation of the stress response [57]. Reduced activation of the prefrontal cortex and/or ventral subiculum is likely to reduce corticolimbic inhibition of the HPA axis [58,59], perhaps explaining the observed enhancement of stress reactivity in epileptic mice. The role of the basal amygdala in stress regulation is complex. The structure is known to be activated by restraint stress, however, stimulation can lead to either reduced or increased corticosterone secretion [37]. Thus, reduced activation of the basal amygdala nucleus may also contribute to HPA axis hyperactivity.

We did not find a significant difference between epileptic and control mice in the number of Fos expressing cells in the paraventricular nucleus of the hypothalamus (PVN), which is the central driver of the HPA axis. However, it is important to note that Fos protein is an indirect all-or-none reporter of neuronal activation, and is not necessarily expressed at levels proportional to the extent of activity. It is possible that a robust initial activation of the PVN occurs following stress exposure in both control and epileptic mice, occluding any subsequent differences in Fos protein levels between the groups. Moreover, single time point analyses do not assess the strength or persistence of PVN drive, which may differ between SE and non-SE groups. Although widely used as a physiological marker of neuronal activity following acute stress, it is possible that Fos is not as sensitive as other biomarkers in the PVN of our model. Recent data suggest that biomarkers such as FosB/ΔFosB and p-ERK1/2 are useful markers to define the aggravation of seizures in mice, and both present activation patterns that are brain region specific [60,61]. It is also possible that HPA drive induced by SE may enhance the releasable pool of ACTH secretagogues in the median eminence (e.g., CRH and arginine vasopressin), which could increase the amount of pituitary drive per given PVN stimulatory episode. Finally, there are numerous other mechanisms that may drive PVN neurons via parallel alternative signaling pathways.

### Aberrant forebrain circuits in TLE may contribute to endocrine and emotional dysregulation

Abnormalities in the stress response have been noted in patients with TLE [14]. In some patients, evoking an emotional stressful response (i.e. using audio and video recordings) is sufficient to trigger spontaneous seizures [62]. In line with our Fos data, patients with epilepsy demonstrate impairments in the activation and functional connectivity of limbic networks at rest [63–66] and in response to stress [14]. Therefore, hypo-activity of these regions may result in reduced inhibitory control over the HPA axis. Studies in humans also suggest that PFC hypofunction may contribute to the development of major depressive illness [67]. In rodents, BALB/c mice, a strain considered to be stress-sensitive and often used as a model of ‘anxiety-like’ behavior, have reduced Fos activation in the medial prefrontal cortex following restraint stress relative to C57BL/6 mice [55]. This suggests a link between reduced prefrontal Fos and enhanced stress reactivity. Additionally, hypoactivation of the PFC and ventral subiculum may increase amygdala excitability [68], and thereby contribute to the development of anxiety-like behaviors. Therefore, the aberrant recruitment of these circuits may have repercussions at the neuroendocrine and behavioral level in TLE [69].

### Epileptic FVB male mice show increased anxiety-like behaviors, hyperactivity and anhedonia

Emotional abnormalities have been found consistently in the pilocarpine SE model in rats [70] and female mice of several strains [29,71,72]. These findings indicate that enhanced emotionality is a reliable and reproducible consequence of TLE in mice, and suggest that similar mechanisms may underlie emotional dysregulation seen in TLE patients. In this study we performed a battery of behavioral tests on male FVB mice, a strain that shows better survival in the pilocarpine model than the C57BL/6 strain typically used in behavioral studies [22,40]. Similar to epileptic female NMRI mice [72], epileptic male FVB mice showed freezing-like behaviors when exposed to novelty, a behavior that is in contrast with the thigmotaxis observed in the C57BL/6 strain [71]. Although we did not see any convulsive seizures or seizure-like activity (CII/CIII Racine scale) during testing, the possibility that freezing reflects non-convulsive seizures cannot be excluded. However, the consistency of the behavioral data from our study and that performed in NMRI females [72] leads us to favor the interpretation that freezing behavior in response to a novel environment reflects a passive coping behavior consistent with an anxiety-like response [73]. Moreover, epileptic FVB males exhibited hyperactivity during homecage monitoring, suggesting that lack of movement in the open field and elevated plus maze are not related to general hypolocomotion. Hyperactive behaviors have been observed in epileptic female mice [71,72] and rats [74], further suggesting that decreased activity is not linked to locomotor deficits. Thus, the observation of freezing in the open field and elevated plus maze, despite overall hyperactivity, may suggest a potential disruption in the fear-anxiety response.

To assess depression-like behaviors, we employed the forced swim test and the sucrose preference test. These two tests measure two different putative phenotypes, passive coping and anhedonia, respectively, that parallel commonly observed symptoms in patients with major depression. In our experiment, and similar to previous observations [71,72], mice did not exhibit increased passive coping (the adoption of an immobile posture) in the forced swim test relative to controls. In contrast the sucrose preference test revealed an anhedonic response; consistent with a depressive-like phenotype. These observations are consistent with prior studies in epileptic mice [29,75]. Absence of an increased immobility phenotype in the forced swim test, despite the presence of anhedonia, may indicate that circuits disrupted in this epilepsy model do not control coping behavior in this context. Alternatively, impairments in memory and potential hyperactivity, previously reported in epileptic mice, may interfere with the sensitivity of this test [71,72].

The forced swim test was developed as a tool to screen for antidepressant efficacy and to investigate different coping strategies [76]; and not necessarily to directly measure depressive-like behavior. In our study, epileptic mice displayed struggling activity at a greater frequency than non-epileptic control mice while displaying no differences in time spent immobile. Recent optogenetic experiments demonstrate that activation of a subset of neurons located in the medial prefrontal cortex is necessary to drive the transition between active swimming and immobility behaviors in the forced swim test [77]. Thus, it is possible that impaired recruitment of the prefrontal cortex in the context of swim stress leads to impairment in the transition between active and passive behaviors.

The present study does not allow us to discern whether the endocrine, cellular and behavioral abnormalities evident in epileptic mice are due to the initial precipitating injury (SE), the epilepsy itself (recurrent seizures), chronic exposure to excess glucocorticoids or some combination thereof. However, prior work implicates glucocorticoids in the progression of the negative consequences of SE. For example, work in our group demonstrates that treatment with the glucocorticoid receptor antagonist, mifepristone, reduces both HPA axis hyperactivity and SE-related hippocampal pathology [23]. Moreover, infusion of glucocorticoids enhances epileptiform activity in SE mice [78]. Studies in humans and rats have shown that traumatic brain injury (a type of precipitating injury) is associated with the development of HPA axis hyperactivity prior to the development of seizures [79,80] suggesting that the precipitating injury could result in endocrine dysfunction. Similarly, evidence suggests that epilepsy itself, as seen in genetic models, may lead to endocrine dysfunction and behavioral comorbidities [81,82]. Most patients with epilepsy who exhibit psychiatric comorbidities do not have a history of SE, and behavioral dysfunction is evident in epilepsy models without SE. SE, therefore, is not a required element. Questions of causality still remain, however, as the underlying brain dysfunction that caused the epilepsy could directly cause HPA dysfunction, or secondarily produce it via inducing epilepsy. Elucidating the causal relationships among these variables will remain a topic of intense study.
